# Methods for genetic transformation of filamentous fungi

**DOI:** 10.1186/s12934-017-0785-7

**Published:** 2017-10-03

**Authors:** Dandan Li, Yu Tang, Jun Lin, Weiwen Cai

**Affiliations:** 1 Institute of Apply Genomics, Fuzhou University, No.2 Xueyuan Road, Fuzhou, 350108 China; 2 School of Basic Medical Sciences, Fujian Medical University, No.1 Xuefubei Road, Fuzhou, 350122 China; 3 College of Biological Science and Engineering, Fuzhou University, No.2 Xueyuan Road, Fuzhou, 350108 China; 4 Triplex International Biosciences (China) Co. LTD, Xiamen, 361100 China

**Keywords:** Filamentous fungi, Protoplast-mediated transformation, *Agrobacterium*-mediated transformation, Electroporation, Biolistic method, Shock-wave-mediated transformation

## Abstract

Filamentous fungi have been of great interest because of their excellent ability as cell factories to manufacture useful products for human beings. The development of genetic transformation techniques is a precondition that enables scientists to target and modify genes efficiently and may reveal the function of target genes. The method to deliver foreign nucleic acid into cells is the sticking point for fungal genome modification. Up to date, there are some general methods of genetic transformation for fungi, including protoplast-mediated transformation, *Agrobacterium*-mediated transformation, electroporation, biolistic method and shock-wave-mediated transformation. This article reviews basic protocols and principles of these transformation methods, as well as their advantages and disadvantages.

## Background

Fungi exist widely in different environments, such as soil, biological wastes and plants. Some of them have been utilized by humans for over 1000 years. In nature, fungi play a vital role in numerous degradation processes. In agriculture, many species of fungi are used for control of plant pests and diseases [1, 2]. In medicine, fungi are utilized to produce antibiotics for the treatment of diseases. For example, penicillin is a product of *Penicillium chrysogenum* and cephalosporin of *Cephalosporium acremonium*. Due to their good capacity in manufacturing valuable proteins and secondary metabolites, fungi are important economic contributors. With the explosion and exploration of fungal genomic sequence information, mycology is coming into a new era of functional studies [3].

Filamentous fungi, molds, grow well and rapidly on simple and inexpensive media and thus are preferred cell factories due to their outstanding capacity in expression and secretion of heterologous proteins with post-translational processing. They have also been used in the production of a wide variety of products, such as citric acid, kojic acid and other organic acids, secondary metabolites like penicillin, cephalosporin, as well as cellulase, amylase, glucanase, rennet, lipase, laccase and unsaturated fatty acids, soy sauce, fermented soya beans.

The development of genetic transformation techniques is a breakthrough in our attempt to genetically modify fungal strains. This technique enables scientists to target and modify genes efficiently to reveal functions of targeted genes, or to insert new genetic elements into the genome of the strain such as promoters to modify the expression of endogenous genes [4].

Conceptually, fungal biotechnology is expected to be transformed by the application of genetic transformation technologies. In practice, genetic transformation of fungi meets with many difficulties. First, although many articles of fungal transformation have been published each team established their own protocols without sufficient details. Second, because of the huge number of fungal species and their complex cell wall structures, different transformation methods are required for different fungal species. Thus, species specific transformation protocols must be optimized for each strain. Even completely new methods must be established for efficient transformation of some strains [5].

In this review, we summarize all major methods of genetic transformation for fungi, including the protoplast-mediated transformation method and the *Agrobacterium*-mediated transformation method, which are common methods used for many years, the electroporation method and the biolistic method, which are not currently popular but are particularly suitable in some specific circumstances, and the emerging shock-wave-mediated transformation technology.

## Common filamentous fungi and their applications

*Aspergillus niger* is a typical species used for producing glucoamylase and citric acid. In recent years, using fungi, such as *A. niger*, to produce cellulase to degrade inexpensive cellulosic materials into glucose with high efficiency has become one of research hotspots. *Aspergillus oryzae,* with a safe application history of over 1000 years, can be applied in producing protease, amylase, glucoamylase, cellulose, and phytase. As pointed out by Kubodera [6], *A. oryzae*, which has been one of the most important workhorses in Japanese fermentation industry, was used for the production of tempeh, one of the oldest fermented soy products in China [7]. *A. oryzae* can secrete a variety of enzymes, including amylase, protease and esterase, etc. Protein in beans can be hydrolyzed into soluble nitrogenous compounds by protease hydrolysis in the fermentation process. In addition, Kojic acid, which has an antibacterial effect on aerobic microbes, is an organic acid produced by *A. oryzae.* Because of its nontoxic property, Kojic acid has important applications such as being used as food additives, or in cosmetics, pharmaceuticals, etc. [8]. *Aspergillus nidulans* has been one of the most widely studied species in terms of genetics and biochemistry [9, 10]. It is often used as model organism in the identification of gene function and protein interaction studies [11, 12]. *Penicillium* is one of the most widely distributed fungi in nature. In medicine, penicillin also is the earliest clinical application of antibiotics. Additionally, *Penicillium* species have the capability of degrading lignocellulose. *Monascus purpureus Went* is mainly used traditionally for making wine, vinegar, food coloring, and meat preservation. In 1979, Brooklyn K (Monacolin K), an agent with activity to lower cholesterol levels [13], was isolated from *Monascus*.

## Transformation methods

The establishment of genetic transformation systems has enabled scientists to transform foreign DNA into filamentous fungi and thus obtained the desired strains for industrial purposes. We now can take full advantage of the superior secretory power of fungi and their excellent efficiency in manufacturing valuable metabolites.

### Protoplast-mediated transformation (PMT)

PMT is the most commonly used fungal transformation method, which relies on a large number of competent fungal protoplasts. The principle is to use some commercially available enzymes to remove fungal complex cell wall components for generating protoplasts. Subsequently, some chemical reagents (such as PEG) are used to promote the fusion of exogenous nucleic acids and protoplasts, as described in more detail below. The components of the fungal cell wall are highly variable among different strains. Even components of the spore coat are significantly different from that of hyphae from the same strain [14]. Thus, there is no universal transformation method that can be applied to different fungal strains. Preparation of protoplast can hardly be standardized. Part of the difficulties comes from our limited knowledge of cell wall hydrolases. Development of an optimized PMT method for fungi still requires significant effort.

PMT is a routinely used transformation method. The method has been under constant improvement to achieve higher efficiency for genetic transformation and for targeting suitable gene loci through gene editing. Preparation of protoplasts requires removing the cell wall, which is mainly achieved through enzyme treatment. Non-enzyme methods to prepare protoplasts have also been reported, such as physical methods including grinding and supersonic wave shock [15]. However, they are not widely used because of practical inconvenience and the low yield of protoplasts. A summary of protoplast-mediated transformation protocols for different fungal species are provided in Table 1.

**Table 1:** Summary of protoplast-mediated transformation protocols for different fungal species

Strain	Starting material	Number of protoplasts	Name of vectors	The concentration of DNA	Selective markers	Transformation efficiency (transformants/μg DNA)	References
*Trichoderma reesei* QM 9414 and VTT-D-79125	Mycelium	5 × 10^7^ to 5 × 10^8^	pSa143 containing the *A. nidulans* argB gene	2–5 μg of transforming DNA	*argB*	600 transformants	[16]
*T. reesei* QM 9414	Mycelium	5 × 10^7^–10^8^	pRLM_EX_30 (5.5 kb)	1–6 μg of transforming DNA	*hygB*	1800–2500 transfomants	[17, 18]
*N. crassa* 74A	Mycelium	1 × 10^7^	pVK88	60 μg/mL	*qa*-*2* ^+^	5-30 transformants	[19]
*A. oryzae* NRRL 492	Mycelium	2 × 10^6^	pILJ16	10 μg	*argB*	5–10 transformants	[20, 21]
*M. purpureus* NRRL 1596, *M. ruber* NRRL 1597 and *M. purpureus* IBCC1	Mycelium	1.8 × 10^8^	pULJL43 (4.6 kb)	15 μg	*Sh ble*	*M. purpureus* NRRL 1596 (870 transformants), *M. ruber* NRRL1597 (137 transformants) and *M. purpureus* IBCC1 (43 transformants)	[22]
*Aspergillus giganteus* IfGB15/0903	Germinated conidia	1 × 10^7^ to 2.5 × 10^8^	pAN7-1	10 μg	*hygB*	55 transformants per 1 × 10^7^ protoplasts	[23]

### Basic steps of the PMT method

**Fig. 1:**
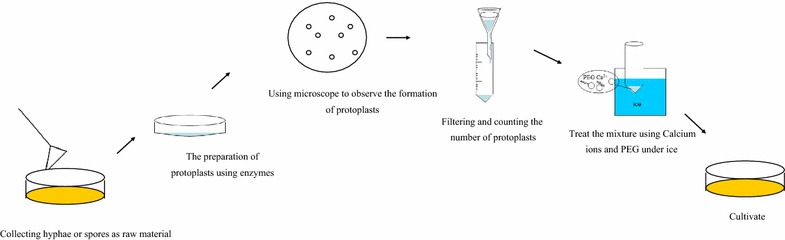
Basic steps of the protoplast-mediated transformation

PMT was first applied to *Saccharomyces cerevisiae*. Researchers [24] prepared protoplasts with the commercial snailase, and used sorbitol to preserve protoplasts. Later, such method was applied to filamentous fungi, such as *Neurospora crassa* [19], and *A. nidulans* [25]. Although the transformation methods have been improved, the basic steps remain essentially the same. Basic steps of the PMT method are provided in Fig. 1.

### Preparation of the protoplasts

The first step in protoplast preparation is the removal of cell wall through enzymatic digestion. The fungal cell wall is comprised of glucan, mannan and chitin. The structure of the fungal cell wall is highly dynamic, and the cell wall varies during the cell division and growth of fungi, as well as in spore germination, hyphal branching and formation of the diaphragm. The cell wall components are also different in different fungal species, therefore, various enzymes should be used in combination. It has been reported that the selection of an appropriate enzyme mix is a key factor in protoplast preparation [26].

In general, the hyphae are sensitive to a suitable enzyme which hydrolyzes its cell wall during the logarithmic phase. In the PMT procedure of *Neurospora*, the protoplasts are prepared by hydrolyzing the newly born hyphae (culture for 4–6 h under 25–30 °C) [27, 28]. Similarly, protoplasts can also be prepared with conidiospores. For example, for *Aspergillus* and *Penicillium*, one can choose germinal spores or thalli [29].

Protoplasts are sensitive to osmotic pressure, care should be taken to maintain a stable osmotic pressure to keep the protoplasts intact during the enzymolysis of cell walls. Thus, osmotic stabilizers (such as sorbitol, sodium chloride, and potassium chloride) should be included in all of the buffers for protoplast preparation to avoid rupture of cells. For instance, sorbitol solution with a concentration of 0.8–1.2 M is used in the protoplast preparation of *N. crassa* [19], *Aspergillus* sp. [30] and *Trichoderma* sp. [17] to maintain the osmotic stability of protoplasts. A summary of protoplast preparation parameters for some common fungal species are provided in Table 2.

**Table 2:** Summary of protoplast preparation parameters for some common fungal species

Strain	Starting material	Enzyme	Enzymolysis conditions	Osmotic stabilizer	Protoplast yield (protoplasts/mL)	References
*A. niger* K10	NA	Lyophilized snail	Incubate at 31 °C for 3 h on a laboratory reciprocal shaker (78 strokes/min, amplitude 3 cm)	0.7 M NaCl	8.9 × 10^6^	[31]
*A. niger* N583	Mycelium	Lysing enzymes, chitinase griseus and β-glucuronidase	Incubate in a 100 mL glass bottle for 2 h at 37 °C and 130 rpm	0.8 M sorbitol	NA	[32]
*Aspergillus nidulans* A89	Mycelium	Yatalase, Kitalase	Incubate 6 h at 30 °C with constant shaking (60–80 rpm)	1 M sorbitol	NA	[33]
*Aspergillus nidulan*s G191	Mycelium	Novozym 234	Incubate 1.5 h at 30 °C	0.6 M KCl	NA	[34]
*Trichoderma atroviride* T11	Mycelium	Lysing enzymes from *Trichoderma. harzianum*	Incubate at 30 °C for 2 h with shaking at 80 rpm	1 M sorbitol	1–5 × 10^6^	[26]
*Penicillium simplicissimum*	Mycelium	Driselase, caylase, cellulase, enzyme cocktail II from Merck company	Incubate at 30 °C for 20 or 180 min	1.2 M sorbitol	2 × 10^9^	[26]
*Trichoderma reesei* QM 9414	Mycelium	Lysing enzymes from *T. harzianum*	Incubate at 28–30 °C for 2 h	1 M sorbitol	NA	[17]
*Trichoderma reesei* QM 9414 and VTT-D-79125	Mycelium	Novozym 234	Incubate at 28 °C for approx. 1.5 h	1 M and 1.2 M sorbitol	5 × 10^7^ to 5 × 10^8^	[16]
*N. crassa* M246 (qa-2 mutation) and M6-11 (arom-9 mutation)	Mycelium	Proteinase K	Incubate at 1 h at room temperature	1 M sorbitol	1 × 10^7^	[19]
*Aspergillus fumigatus* 2085	Mycelium	β-glucuronidase	Incubate at 37 °C for 1 h with occasional shaking	0.7 M KCl	1 × 10^6^	[35]
*A. oryzae* NRRL 492	Mycelium	Novozyme 234 and β-glucuronidase	Incubate at 30 °C for 1.5 h with shaking at 80 rpm	0.6 M sorbitol and 1 M sucrose	Each 400 mL culture yielded 5–10 × 10^7^ protoplasts	[20, 21]
*Rhizopus oryzae* AS 3.819	Mycelium	Helicase, cellulase, lyticase	Incubate at 35 °C for 140 min with gentle shaking	0.6 M sorbitol	1 × 10^7^	[36]
*Monascus purpureus*	Mycelium	Glucanex, lysing enzyme from *T. harzianum* β-glucuronidase and Caylase	Incubate at 28 °C for 4 h with orbital shaking (100 rpm)	0.5 M KCl and 0.1 M MgSO4	1 × 10^9^	[22]
*Penicillium expansum*	Mycelium	0.6% cellulase and 0.6% snailase	Incubate at 25–35 °C for 3–3.5 h	0.6 M NaCl	2.36 × 10^8^	[37]

### Uptake of exogenous DNA

The solution used to suspend protoplasts usually contains calcium ions and osmotic stabilizers. Calcium is thought to open channels in the cytomembrane, which facilitates entry of exogenous DNA into the cell, while osmotic stabilizer are necessary for maintaining the morphology of the protoplasts. Usually, certain amount of polyethylene glycol (PEG) is added together with purified DNA (which can either be the circular double-stranded DNA or the linearized DNA). PEG is a commonly used cell fusion promoter [38]. It can form the molecular bridge between cells or between cytomembrane and DNA, and thus promotes adhesion. In addition, it can also induce disordered charges on the cytomembrane surface, alter the membrane permeability, and facilitate entry of exogenous nucleic acids into cells [39].

PEG is a crucial agent enhancing transformation efficiency. Low transformation efficiency in most cases can be improved by adding more PEG. Under normal conditions, the performance of low-molecular-weight PEG (like PEG3000) is superior to that of high-molecular-weight PEG (like PEG8000). However, this needs to be optimized for various species [40].

Transformation efficiency is also influenced by temperature. Generally, the DNA and protoplast mixture should be placed on ice for 15–30 min, so that the DNA can adhere to the surface of protoplasts [41].

### Regeneration of protoplasts

In order to guarantee good recovery of viable protoplasts, protoplasts are allowed to recuperate on the plate with no selection pressure for a certain amount before they are transferred to a selective plate. An osmotic stabilizer should be included in the regeneration culture. Stable osmotic pressure is the key factor for protoplast to regenerate cell wall. Only the protoplasts that carry exogenous nucleic acids can grow on the selective medium.

### Comments on the PMT method

Protoplast transformation method is simple and effective with no need for expensive equipment. But the protocol involves many steps and critical reagents. Each step needs to be optimized and the quality of the reagents needs to be critically tested. The growth status of fungi being transformed needs to be carefully monitored. Experience is critical for the successful implementation of this method.

### *Agrobacterium* -mediated transformation (AMT)

*Agrobacterium* is a Gram-negative bacterium commonly found in soil. *Agrobacterium tumefaciens* can infect injured plants. The tumor-inducing plasmid of > 200 kb, which is also referred to as the Ti plasmid, could be isolated in the early stage of infection. When *A. tumefaciens* infects a plant, it enters the plant through the wound, and integrates part of the Ti plasmid into the genome of the infected plant cells. The integrated DNA fragment from the Ti plasmid is commonly referred to as transfer DNA or T-DNA. The T-DNA inserts into the plant genome randomly as monoclone. The T-DNA is flanked by two directional imperfect repeats (called left and right border) and contains genes that encode enzymes responsible for the formation of plant hormones, which cause tumor growth [42]. A binary vector was designed to have the target gene inserted in between the left and right T-DNA borders, and the recombinant plasmid was transformed into the *Agrobacterium tumefaciens*. The positive *Agrobacterium* clone was used as a vehicle to integrate the target gene into the fungal genome. The specific steps will be discussed in detail below.

**Fig. 2:**
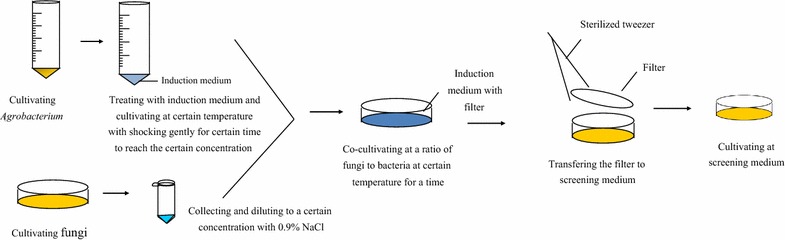
The basic steps of the *Agrobacterium*–mediated transformation

AMT method has been shown to be more stable and efficient than conventional transformation methods since the first paper [43] reported that this method could be applied to fungal transformation. The AMT method was first applied to transform *S. cerevisiae* [28]. A plasmid carrying a hygromycin-resistance gene is commonly used [43] to transform *Aspergillus awamori* [44]. The AMT method has been applied to many *Ascomycetes*, including the *Aspergillu*s [43], and *Monascus purpureus* [22]. The basic steps of AMT method are provided in Fig. 2. A summary of *Agrobacterium*-mediated transformation protocol for different fungal species is provided in Table 3.

**Table 3:** Summary of *Agrobacterium*-mediated transformation protocols for different fungal species

Fungus	*Agrobacterium*	Starting material	The number of fungal cells	Pre-culturing of *Agrobacterium*	The conditions of co-culturing	Name of vector (size)	Selective marker	Transformation efficiency	References
*Aspergillus awamori*	*A. tumefaciens* LBA1100	Conidiospores	1 × 10^6^	Incubate at 28 °C 4–5 h with 0.2 M AS and 100 rpm to OD600 nm of 0.8	Incubate the plates for 3 days at 22.5 °C	NA	*hygB*	200–250 transformants per 10^6^ conidiospores	[45]
*Aspergillus fumigatus* B-5233	*A. tumefaciens* EHA105	Conidia	1 × 10^7^	Further incubation at 28 °C for 24 h to a ratio of 1:10 (conidia to bacteria)	Incubated at 37 °C for 3 days	pDHt/*hph* (NA)	*hygB*	100 transformants per 10^7^ conidia	[46]
*Trichoderma reesei* QM 9414	*A. tumefaciens* AGL-1	Protoplasts and conidia	1 × 10^7^	Further incubation for 6 h at 28 °C to OD600 nm of 0.15	Incubation at 25 °C for 48 h	pBI-hph (15846 bp)	*hygB*	Start from protoplasts: 2000 to 9000 transformants per 10^7^ protoplasts. Start from conidia: 200–500 transformants per 10^7^ condia	[47]
*Mucor circinelloides* ATCC1216b	*A. tumefaciens* AGL-1	Spores	1 × 10^5^ spores per mL and	Incubate for 4 h at 20 °C to OD600 nm of 0.6	Incubation at 15 °C for 6 days	pTiBo542 (NA)	*hygB*	2000–9000 transformants per 10^7^ sporangiospores	[48]
*Aspergillus giganteus* IfGB15/0903	*A. tumefaciens* LBA1100	Germinated conidia	5 × 10^7^	Incubate for 6 h at 30 °C with 200 μM AS to OD 600 nm of 2.5	Incubation at 24 °C for 3 days	pUR5750 (NA)	*hygB*	500 to 7900 transformants per 10^8^ conidia	[23]

#### Factors that influence the AMT efficiency

Many factors affect the AMT efficiency, including the type of starting fungal material (protoplast, spore, hypha, and fruit body tissue), concentration of the acetosyringone, ratio of fungus to *Agrobacterium*, and the condition for co-culturing.

1. *The type of starting fungal material* The AMT method can use the protoplasts, spores, hyphae, and fruit body tissue of fungi as the recipient. Appropriate starting materials should be selected for different strains. For instance, the AMT method only works for the protoplasts of *Rhizopus. oryzae* and *Mucor circinelloides*, while spores or germinal spores would not produce transformants [49].
2. *The concentration of acetosyringone (AS)* AS acts on two stages during the AMT process. One is the induction process, and the other is the transformation process. AS is generally used to induce the expression of the *Vir* domain of T-DNA, and the gene in the *Vir* domain activate the transfer of T-DNA. Numerous studies have demonstrated that an appropriate amount of AS was necessary during the transformation process. However, the addition of AS is not absolutely necessary during the pre-culturing stage of the *Agrobacterium*, which could reduce transformation efficiency for some strains. The concentration of AS is an important factor affecting the transformation efficiency during the fungus-*Agrobacterium* co-culturing process in the AMT of *Aspergillus awamori* [45].
3. *The ratio of fungi to Agrobacterium* Within certain limits, the transformation efficiency will reach the maximum level with the increase of amount of fungus or *Agrobacterium*. An optimal ratio for the AMT for different fungi must be empirically determined. Ratio of fungal to that of bacterial cells should be optimized for different fungus-*Agrobacterium* transformation systems.
4. *The condition for co-culturing* The conditions for co-culturing are an important factor in the AMT method. This includes culture time, temperature, pH, and the selection of filter. The temperature and time for co-culturing are the key factors among the AMT steps. In the fungus-*Agrobacterium* transformation, an appropriate condition to start is a temperature of 20–28 °C and a co-culturing time of 16–96 h. A lower temperature (20–25 °C) is usually beneficial for the AMT method. The filter, which is hydrophilic and serves as support for fungus-*Agrobacterium* co-culturing, facilitates the transfer of single colonies to the screening plate. A nitrocellulose membrane, nylon membrane, filter paper, cellophane and polyvinylidene fluoride (PVDF) membrane can be used as the filter [3].

#### Comments on the *Agrobacterium*-mediated transformation

The AMT method opens up a new avenue for those fungi recalcitrant to transformation by conventional methods. The AMT method is especially suitable for generating knock-in mutations in fungi because T-DNA randomly inserts into the genome as a single copy. In addition, AMT can achieve high homologous recombination efficiency in various gene targeting experiments [3].

Major advantages of the AMT method include: firstly, diversified transformation recipients, including protoplasts, hyphae, and spores; secondly, the ability to integrate exogenous genes into the genome to form stable transformants; and thirdly, high transformation efficiency resulting in a large number of transformants [3].

The AMT method requires binary vectors, which are tedious to prepare. Multiple factors need to be taken into consideration in the optimization of the transformation process. This is a major limitation of the AMT method [50, 51].

### Electroporation transformation

Electroporation is a simple, rapid and efficient transformation method for filamentous fungi. In electroporation, electric charges are stored in a capacitor to build a high voltage, the sample is struck by the impulse voltage, and the exogenous nucleic acid can be transferred instantly into cells. Usually, square waves or exponential decay waves are used in the transformation of fungi [52, 53]. Exponential-decay pulses are generated simply by charging and discharging a capacitor. The electric field declines exponentially from the peak value. A square wave is a non-sinusoidal periodic waveform (which can be represented as an infinite summation of sinusoidal waves), in which the amplitude alternates at a steady frequency between fixed minimum and maximum values. Different waveforms of electroporation are utilized for different species. A summary of waveforms used in electroporation for different species is provided in Table 4.

**Table 4:** Summary of waveforms used in electroporation of different species

Strain	Waveform	References
*Saccharomyces cerevisiae*	Exponential decay waveform	[54]
*Pichia pastoris*	Exponential decay waveform	[55]
*A. niger* ATCC 20739	Exponential decay waveform	[52]
*Rhizopus oryzae*	Square wave	[53]
*Aspergillus nidulans*	Exponential decay waveform	[56]
*Trichoderma harzianum*	Exponential decay waveform	[57]

When a cell is exposed to the electric field, the structure of the cytomembrane will be changed with a voltage induced between the cytomembrane. Micropores can be formed in the cytomembrane after electric shock. The induced cell wall permeability is reversible within the thresholds of the voltage and the duration, otherwise, it will cause irreversible injury to the cells. Therefore, the micropores in the cytomembrane appear to have two patterns after electric shock, the reversible and the irreversible pattern. The lipid and protein molecules in the cytomembrane can restore the original structure when an appropriate field intensity is applied, while the irreversible electric shock will give rise to irreparability or extremely slow recovery, which eventually leads to cell death [58]. Exogenous DNA can be transferred into the bacterium [59], plant protoplast [60], animal cell [61] and filamentous fungi [62] through electroporation. This method has been successfully applied to multiple fungi. Ozeki et al. discovered that germinal spores are more amenable to transformation by electroporation [52]. In recent years, electroporation has become a reliable method for gene transformation of some common strains [63]. A summary of electroporation-mediated transformation protocols for different fungal species is provided in Table 5.

**Table 5:** Summary of electroporation-mediated transformation protocols for different fungal species

Strain	Waveform	Instrument	Electroporation parameters	Raw material	Number of cell	Name and size of vectors	Amount of DNA	Selective markers	Transformation efficiency (per μg DNA)	References
*Rhizopus oryzae* AS 3.819	NA	Multiporator (Eppendorf)	12 kV/cm	Protoplasts	1 × 10^7^	pBS-hygro-*ldh*A (8533 bp)	5 μg	*hygB*	10.2 transformants	[36]
*R. oryzae* AS 3.819	NA	Multiporator (Eppendorf)	15 kV/cm	Germinated spores	1 × 10^7^	pBS-hygro-*ldh*A (8533 bp)	5 μg	*hygB*	8.8 transformants	[36]
*A. niger* ATCC 20739	Exponential decay waveform	Electro Gene Transfer Unit	6 kV/cm and 3 ms time constant	Germinated spores	1 × 10^7^/0.4 mL	pXbal92 (5.7 kb) and pBXba2 (6.0 kb)	1–10 μg	*argB* ^−^	1.2 transformants for integrative vector and 100 colonies for plasmid DNA.	[52]
*Penicillium urticae* NRRL 2159A	Exponential decay waveform	Gene Pulser (Bio-Rad) Multiporatr (Eppendorf)	12.5 kV/cm, 8.6 ms time constant and 25 μF	Germinated conidia	8 × 10^6^	pCSN44(NA)	1–5 μg	*hygB*	1.8 × 10^3^ transformants	[64]
*A. oryzae* ATCC 14895	Exponential decay waveform	Gene Pulser (Bio-Rad) Multiporatr (Eppendorf)	11–12.5 kV/cm, 4.6–4.8 ms time constant and 25 μF	Germinated conidia	2.5 × 10^6^	pBEN(NA)	1–5 μg	Benomyl^R^	2.6 × 10^3^ transformants	[64]
*Leptosphaeria maculans* “Virulent”	Exponential decay waveform	Gene Pulser (Bio-Rad) Multiporatr (Eppendorf)	12.5 kV/cm, 4.8 ms time constant and 25 μF	Germinated conidia	1.2 × 10^6^	pCSN44(NA)	1-5 μg	*hygB*	1–6 × 10^2^ transformants	[64]
*N. Crassa* R-206A	Exponential decay waveform	Gene Pulser (Bio-Rad) Multiporatr (Eppendorf)	12.5 kV/cm, 5 ms time constant and 25 μF	Germinated conidia	3–6 × 10^6^	Bsqa(NA)	1–5 μg	*hygB*	5.7 × 10^3^ transformants	[64]

### Factors that influence electroporation transformation

#### Electroporation parameters

1. *Electric field intensity* Electric field intensity is the most important factor that influences the electroporation efficiency. When the applied electric field intensity reaches the magnitude of kV/cm and the pulse width of μs–ms scale, the cytomembrane will be changed and many micropores will be generated on the cell walls [65]. High electric field intensity is associated with high uptake rate of exogenous nucleic acids and lower cell survival rate. However, various types of cells require different electric field intensities due to differences in cytomembrane components [66]. Few micropores are formed when the electric field intensity does not exceed the required threshold. In the contrary, excessive electric field intensity will result in irreversible damage to the cytomembrane, leading to cell death [58].
2. *Capacitance* During the electroporation process, variation in electric charges and the electric field intensity applied to the cell suspension depend on the capacitance and the pulse duration. The intensity and duration of the pulse are also influenced by the capacitance, therefore, greater capacitance has better transformation effects [67].
3. *Pulse duration and frequency* The duration of perforation on the cytomembrane, which directly related to electroporation transformation efficiency, is influenced by pulse duration and frequency [68].

### Electroporation environment and external factors

1. *Buffer solution* The buffer solution provides an important environment for electroporation of cells, and the pH value of the electric shock buffer solution is of great importance. Normally, a buffer of pH 7.0 is used. Cells are easily punctured and killed at pH higher than 7.0 [52].
2. *Temperature* A large amount of heat is produced during the electroporation process, which will be released into the buffer solution. Therefore, a reduced temperature (0–4 °C) is recommended for better effect [66]. Furthermore, ice-bathing the pre-electric-shock mixture can also improve the electric shock efficiency.
3. *Concentration of exogenous nucleic acid* Overall, the electroporation efficiency increases with the concentration of exogenous nucleic acid. Compact superhelix DNA more easily enters the cells through the cytomembrane. In 1995, one study reported that every 1 μg of plasmid DNA could generate 100 transformants for *A. niger* [52].

#### Comments on the electroporation method

The electroporation method has been extensively applied to numerous types of cells, including prokaryotes and eukaryotes. This technology has the potential to be the method of choice for the transformation of unexplored fungal species. Compared with the PMT method, where complicated steps are involved, electroporation is simple and more convenient. However, the mechanism of electroporation still remains unclear. The perforation rate of the cytomembrane is dependent on many parameters of the electric field. And also, it requires suitable buffer conditions to be optimally effective.

### Biolistic transformation

Biolistic transformation is also known as particle bombardment. Its principle is that foreign DNA is adsorbed on the surface of tungsten or gold particles. Under the push of high pressure, the particles are injected into host cells. Particle bombardment can realize both stable and transient transformation.

Various factors affect the efficiency of bombardment in patterns of complex interactions [69]. Biological parameters (cell type, growth condition, and cell density) and instrumental settings (particle type and size, vacuum and pressure level, target distance) are important variables [70].

Particle bombardment is the most powerful among all the genetic transformation methods. It is not subject to the limitations of cell types of host or species. For fungi, particle bombardment is sufficiently efficient for those organisms that are difficult to culture or from which protoplasts are hard to prepare. Particle bombardment is easy and convenient to operate. However, instruments and consumables for particle bombardment are expensive. It will only be considered in the case where other methods fail to work. At present, particle bombardment has been utilized to successfully transform *A. nidulans* and *T. reesei* [71, 72], etc.

### Shock-wave-mediated transformation (SWMT)

SWMT utilizes the principle of energy transformation and transmission to generate transient pressure disturbance and twisting force across cells to form transient cavitation effect [73]. This method has been applied in medical treatment such as orthopedics and crushing kidney stones [74, 75]. SWMT changes the permeability of cell membranes through acoustic cavitation resulting in the uptake of exogenous nucleic acid into cells. The method has been successfully used in introducing exogenous nucleic acid into *Escherichia coli*, *Pseudomonas aeruginosa* and *Salmonella typhimurium* [76–78]. In 2013, Denis Magaña-Ortíz et al. first reported the application of SWMT for fungi, including *A. niger*, *Fusarium oxysporum*, and *Phanerochaete chrysosporium* [79]. In this article, three advantages of the SWMT method were noted. Firstly, compared with conventional transformation methods, this method is able to directly act on spores but not protoplasts. Secondly, the physical parameters were easily controllable, only the number of spores, energy and speed of the shockwave needed to be precisely controlled. Thirdly, the transformation efficiency was excellent. Results of Denis Magaña-Ortíz et al. indicated that, compared with the *Agrobacterium* transformation method, SWMT method could enhance the transformation efficiency by 5400 fold for *A. niger* [79].

But some limitations in this transformation method were also notable. Since a large proportion of DNA is damaged in the shock wave treatment, the transformation efficiency as determined by the ratio of DNA to cells was quite low [80]. However, for the number of cells involved, the transformation efficiency was significantly higher [52, 81]. In evaluating the efficiency, one needs to consider two aspects: the amount of DNA and the number of cells. For example, in the experiment performed by Magana-Ortiz et al. [79], in general, plasmid DNA used in protoplast transformation and electroporation is about 1–10 μg [16–19, 23, 36, 52, 64]. It is expensive and inconvenient to produce such huge amount of plasmid in the laboratory for the SWMT method. Furthermore, shockwave sources and instruments are expensive because they are primarily designed for medical purpose. This turns out to be a major obstacle to adopting this method in a microbiology laboratory with limited resources.

## Prospects

Filamentous fungi are a category of organisms widely existing in nature. Their secondary metabolite products are used in all aspects of our daily life. Their important economic value is self-evident. However, although filamentous fungi can produce beneficial secondary metabolites, some filamentous fungi also produce secondary metabolites that are toxic to humans and animals, such as mycotoxins. Mycotoxins seriously threaten the health and safety of livestock, poultry, and even rodents and humans. Most mycotoxins have strong acute toxicity. Some types of mycotoxins are strong carcinogens and mutagens and can damage liver, kidney, and other organs [82]. With the development of the genetic transformation method for fungi, these toxic genes could be removed and fungi can be made much safer or even harmless to humans and animals.

Transformation technology is a premise for modifying the genome of filamentous fungi. At present, the PMT method, electroporation transformation method and AMT method are mainly utilized in the study of fungal transformation. Compared with electroporation transformation, the protocols of PMT or AMT have been well developed and routinely used. At present, no universal fungal transformation methods works for every fungal species and one must find a specific protocol for the species of interest. In mammalian cell research, some fast, effective and simple transformation methods such as viral vectors-mediated transformation, nanomaterial-mediated transformation or liposome-mediated transformation are well established. Unfortunately, these methods are not suitable for fungi. There has been no significant breakthrough in fungal transformation for almost over 20 years.

In recent years, genome editing technology has been rapidly developed. The existing technologies mainly include Zinc-finger nucleases (ZFNs) [83, 84] technology, transcription activator-like effector nucleases (TALENs) [85] and clustered regularly interspersed short palindromic repeats (CRISPR) technology [86]. ZFNs have been widely used, but may results in a high off-targeting rate and high level of cytotoxicity. TALENs is similar but superior to ZFNs, which is much more cumbersome and expensive [87]. The combination of fungal transformation technology and effective genome editing technology are expected to realize the modification of fungal genomes to improve the protein production capacity of industrial fungi, to reduce the level of mycotoxins of certain strains, and to develop new strains for protein expression. However, application of genome editing technology to filamentous fungi is currently at an early stage. Numerous issues await exploration.
